# Pattern and severity of early childhood caries in Southern Italy: a preschool-based cross-sectional study

**DOI:** 10.1186/1471-2458-14-206

**Published:** 2014-02-27

**Authors:** Carmelo GA Nobile, Leonzio Fortunato, Aida Bianco, Claudia Pileggi, Maria Pavia

**Affiliations:** 1 Department of Health Sciences, Medical School, University of Catanzaro “Magna Græcia”, Via T. Campanella, 115, Catanzaro 88100, Italy

**Keywords:** Early childhood caries, Public health, Feeding habits, Children, Prevention

## Abstract

**Background:**

This survey was intended to investigate prevalence and severity of early childhood caries (ECC) in a sample of children in Southern Italy and to identify factors that may be related to this condition.

**Methods:**

The study was designed as a cross-sectional survey. The study population (children aged 36–71 months) attending thirteen kindergartens was randomly selected through a two-stage cluster sampling procedure. Parents/guardians of all eligible children were invited to participate filling out a structured self-administered questionnaire, and after having returned the informed consent form an oral examination of the child was performed at school. The questionnaire included information on: socio-demographics about parents/guardians and child, pregnancy and newborn characteristics, oral hygiene habits of child, eating habits particularly on consumption of sweets, access to dental services, and infant feeding practices. The WHO caries diagnostic criteria for deciduous decayed, missing and filled teeth (dmft) and surfaces (dmfs) were used to record ECC and severe-ECC (S-ECC). Univariate and multiple logistic regression analyses were conducted to evaluate statistical associations of social demographics, infant feeding practices, oral hygiene habits, and access to dental services to ECC, S-ECC, dmft and dmfs.

**Results:**

515 children participated in the study. 19% had experienced ECC, and 2.7% severe-ECC (S-ECC), with a mean dmft and dmfs scores of 0.51 and 0.99, respectively. Mean dmft was 2.68 in ECC subjects, and 6.86 in S-ECC subjects. Statistical analysis showed that prevalence of ECC significantly increased with age (OR = 1.95; 95% CI = 1.3-2.91) and duration of breastfeeding (OR = 1.26; 95% CI = 1.01-1.57), whereas it was significantly lower in children of more educated mothers (OR = 0.64; 95% CI = 0.42-0.96), and higher in those who had been visited by a dentist in the previous year (OR = 3.29; 95% CI = 1.72-6.33).

**Conclusions:**

Results of our study demonstrate that even in Western countries ECC and S-ECC represent a significant burden in preschool children, particularly in those disadvantaged, and that most of the known modifiable associated factors regarding feeding practices and oral hygiene are still very spread in the population.

## Background

Although largely preventable and despite the significant improvement of oral health in the past few decades, dental caries remains the most common chronic disease among children and adolescents [1], both in developed and developing countries.

Considering the potential negative impact on overall oral health and on oral health related quality of life of children aged 2–5 years and their parents [2], the early occurrence of dental caries during lifetime, namely early childhood caries (ECC), is of particular concern, since it represents an indicator of preventive missed opportunities, also in developed and developing countries. Moreover, it has been reported that ECC is particularly concentrated in socially disadvantaged children and it has been described as a social, political, behavioral, medical, psychosocial, economical and dental problem [3].

Many definitions of ECC have appeared in the literature, complicating comparisons among different studies and populations. The American Academy of Pediatric Dentistry has then defined ECC as “the presence of 1 or more decayed (noncavitated or cavitated lesions), missing (due to caries), or filled tooth surfaces in any primary tooth in a child 71 months of age or younger” [4]. They also defined severe ECC (S-ECC) with differences according to age: in children younger than 3 years of age, it is defined as any sign of smooth-surface caries, whereas in children from 3 to 5, it is defined as 1 or more cavitated, missing (due to caries), or filled smooth surfaces in primary maxillary anterior teeth, or a decayed, missing or filled score (dmft) ≥ 4 at age 3, or a dmft ≥ 5 at age 4, or a dmft ≥ 6 at age 5 [4].

Prevalence of ECC varies among populations, and, within the same population, according to several established or controversial associated factors. It is well established that dietary and biological factors such as frequent consumption of high sugar foods and the presence of bacteria such as *Streptococci mutans*[5] contribute to ECC development; however ‘cariogenic’ parental practices are less well understood. Indeed, among parental socio-demographic factors, being of lower social class [6,7], having lower family income [8,9], and living in a single-parent family [10] are contributing factors in the development of dental caries. Education level [11] and living conditions [12] also reflect socio-economic status and may affect knowledge and skills for making health behavior choices and can influence access to health resources. Minority ethnicity [13-15], or immigrant children [6,16], have been found to be at higher risk of caries. Also child feeding practices are important in the early establishment of long term dietary behaviours [17] and short-term development of ECC [18], although controversies still exist. Some studies identified both bottle feeding [19,20] and breastfeeding [21,22] as ECC risk factors, whereas other studies found no effect for either breastfeeding [23-25] or bottle feeding [26] or differences between the two feeding practices [27]. It is also possible that the length of breast and bottle feeding contributes to caries development. Delayed weaning (e.g., between 12 and 36 months) has been found to be associated with caries in various studies [12,28]. However, other studies reported no effect for age at which child was weaned from breast [23,29,30] or bottle [24]. Children who received nocturnal bottle feeding had higher risk for ECC [19,20,27]. However, nocturnal breastfeeding, [31] its frequency, particularly if more than twice throughout the night [29,31] and duration > 15 minutes [31] appear to be risk factors. However other studies did not find an association between nocturnal feeding [20,32,33] and caries. Delayed introduction to toothbrushing (e.g., after 12 months of age) is somewhat controversial. Frequency of brushing, [12,14,33] bedtime brushing [33] and time spent brushing [14] were found to be associated with ECC in some studies, however other studies failed to find associations between frequency of toothbrushing and caries [24,26,30,32].

Considering the enormous opportunities of prevention of ECC, effort in the investigation of pattern and severity of ECC, pathways of maladaptive behaviours and poor health outcomes in children are important requisites that can be used to inform the development of evidence-based prevention interventions that target those most at risk.

Although many oral surveys have been conducted to explore the oral health of subgroups of population [34-37], few attention has been posed to ECC and S-ECC.

The primary purpose of this study was to investigate prevalence and severity of ECC; a secondary goal was to identify the extent to which well established or more controversial factors are associated to ECC.

## Methods

### Study design

The study was designed as a cross-sectional survey. The study population was selected through a two-stage cluster sampling procedure. Thirteen out of 41 kindergartens in the city of Lamezia Terme (CZ) in Southern Italy were randomly selected, and all children aged 36–71 months attending these schools were considered eligible. No exclusion criteria of children and parents were established. Parents/guardians of all eligible children were informed through a letter about the purposes and the main issues of the study and invited to participate. The participation consisted, after having returned an informed consent, in the compilation of a structured self-administered questionnaire by the parent/guardian and on an oral examination of the child performed at school.

The sample size was calculated before study initiation, assuming that ECC prevalence was about 20% in accordance with the literature, a margin of error of 5%, and a 95% confidence level. Consequently, a sample of 246 children was sought. We decided to be conservative and to inflate our sample size to 640 children by anticipating a response rate of 40%.

### Survey instruments

The questionnaire completed by parents/guardians included information on: socio-demographics about parents/guardians (sex, age, education level) and child (sex, age, birth order, number of brothers/sisters), pregnancy and newborn characteristics (age of mother at delivery, gestational age, birth weight), oral hygiene habits of child (age at first and frequency of toothbrushing, supervision of toothbrushing, use of toothpaste, use of fluoride supplements), eating habits particularly on consumption of sweets, access to dental services (dental problems and visits in the previous year, reasons for dental visits and treatments received), and infant feeding practices (occurrence and duration of breastfeeding, bottle feeding, sleep with a sweetened bottle and/or pacifier, non-nutritive sucking habits with fingers and pacifiers, and age at start using cup).

### Oral examinations

The dental examinations were conducted at school by one trained and calibrated dentist (LF). Duplicate clinical examinations were performed to test intra-examiner reliability until substantial correlation measured by Cohen’s Kappa (k ≥ 0.6) and a >90% Kappa agreement were shown.

Children were seated on a chair facing a window and the examiner used portable equipment consisting of plane mouth mirror, explorer, and a periodontal ball-pointed probe. No radiographs were taken. The WHO caries diagnostic criteria for dmft and dmf surfaces (dmfs) [38] were used to record ECC and S-ECC. The prevalence of natural teeth was recorded clinically according to the WHO criteria.

The Ethics Committee of the “Mater Domini” Hospital of Catanzaro (Italy) approved the protocol of the study (Prot. EC no.73/2006).

### Statistical analysis

Univariate analysis was conducted using appropriate tests (*χ*^*2*^ and *χ*^*2*^ for trend, Fisher exact test, t-test, Anova) to evaluate associations of social demographics, infant feeding practices, oral hygiene habits, and access to dental services to ECC, S-ECC, dmft and dmfs. Variables that showed significant associations and potential confounders were included in multiple logistic regression models, that were constructed to assess the independent effect of these covariates on prevalence of ECC (Model 1) and S-ECC (Model 2). The variables entered in the models were the following: child’s age (months) (ordinal: 0 = 36-47; 1 = 48-59; 2 = 60-71), mother’s age (years) (ordinal: 0 = ≤30; 1 = 31-35; 2 = 36-40; 3 = ≥41), mother’s education level (ordinal: 0 = none/elementary school; 1 = junior high school; 2 = high school; 3 = university), maternal age at delivery (years) (ordinal: 0=≤25; 1=26-30; 2=31-35; 3=≥36), history and duration of breastfeeding (months) (ordinal: 0 = no; 1 = ≤4; 2 = 5-10; 3 = 11-19; 4 = ≥20), sleep with sweetened bottle or pacifier (0 = no; 1 = yes), start using cup (months) (ordinal: 0 = ≤12; 1 = 13-18; 2 = ≥19), start toothbrushing (months) (ordinal: 0 = ≤12; 1 = 13-24; 2 = ≥25), dental visit in the previous year (0 = no; 1 = yes).

All analyses were conducted using the Stata version 11 software program [39].

## Results

Of the total 640 eligible children, 515 participated in the study, thus giving a response rate of 80.5%. Non-responses were due to no-contact (i.e. children were not at the kindergarten when the research team attempted to make first and second contact), not-able (i.e. contacted parents wanted to participate but children were ill at the time of first and second oral examinations), and refusal (i.e. contacted parents refused to answer to the questionnaire for concern of oral examinations or privacy reasons). Children were evenly distributed between males and females, 161 were 3, 196 were 4, and 158 were five years old. Overall, 98 (19%) had experienced ECC, and 14 (2.7%) S-ECC, with a mean dmft and dmfs of 0.51 (SD±1.4) and 0.99 (SD±3.04), respectively. Among those who had ECC, 97% had untreated decayed teeth. Mean dmft was 2.68 (SD±2.11) in ECC subjects, and 6.86 (SD±1.56) in S-ECC subjects. ECC pattern and severity according to social demographics, pregnancy and newborn characteristics, infant feeding practices, oral hygiene and eating habits, and access to dental services are reported in Table 1.

**Table 1:** ECC pattern according to newborn characteristics, feeding practices, oral hygiene habits, and access to dental services

**Variable or practice**			**ECC**		**S-ECC**		**dmft Mean ± SD**	**dt Mean ± SD**	**dmfs Mean ± SD**	**ds Mean ± SD**
	**N**	**%**	**N**	**%**	**N**	**%**				
***Social demographics and newborn characteristics***										
Age, months										
*36-47*	161	31.2	14	8.7	2	1.2	0.17 ± 0.78	0.17 ± 0.78	0.30 ± 1.65	0.25 ± 1.6
*48-59*	196	38.1	37	18.9	6	3.1	0.49 ± 1.38	0.45 ± 1.29	0.96 ± 3.09	0.86 ± 2.98
*60-71*	158	30.7	47	29.8	6	3.8	0.89 ± 1.78	0.86 ± 1.77	1.71 ± 3.81	1.68 ± 3.81
			*χ*^*2*^ for trend = 22.89; p < 0.001		Fisher’s exact p = 0.324		F = 10.99 p < 0.001	F = 10.86 p < 0.001	F = 8.78 p < 0.001	F = 9.40 p < 0.001
Gender										
*Male*	262	50.9	54	20.6	8	3.1	0.58 ± 1.51	0.54 ± 1.46	1.12 ± 3.24	1.03 ± 3.18
*Female*	253	49.1	44	17.4	6	2.4	0.44 ± 1.27	0.43 ± 1.25	0.85 ± 2.82	0.81 ± 2.78
			*χ*^*2*^ = 0.87; p = 0.352		*χ*^*2*^ = 0.23; p = 0.634		t-test = -1.15; p = 0.251	t-test = -0.923; p = 0.355	t-test = -1; p = 0.317	t-test = -0.85; p = 0.395
Father’s age, years										
*≤35*	177	35.1	36	20.3	5	2.8	0.6 ± 1.57	0.6 ± 1.57	1.24 ± 3.72	1.2 ± 3.71
*36-40*	160	31.7	31	19.4	7	4.4	0.52 ± 1.37	0.44 ± 1.25	1.04 ± 2.88	0.86 ± 2.72
*41-45*	125	24.7	23	18.4	2	1.6	0.46 ± 1.35	0.46 ± 1.35	0.78 ± 2.63	0.78 ± 2.63
*≥46*	43	8.5	8	18.6	0	-	0.37 ± 0.95	0.37 ± 0.95	0.56 ± 1.58	0.56 ± 1.58
			*χ*^*2*^ for trend = 0.18; p = 0.676		Fisher’s exact p = 0.446		F = 0.45; p = 0.714	F = 0.6; p = 0.618	F = 0.87; p = 0.458	F = 0.81; p = 0.486
Mother’s age, years										
*≤30*	105	20.7	32	30.5	9	8.6	1.02 ± 2.12	0.91 ± 2.02	1.91 ± 4.91	1.81 ± 4.86
*31-35*	166	32.7	24	14.5	1	0.6	0.3 ± 0.86	0.3 ± 0.86	0.71 ± 2.07	0.61 ± 1.95
*36-40*	171	33.6	31	18.1	4	2.3	0.49 ± 1.39	0.49 ± 1.39	0.91 ± 2.73	0.87 ± 2.7
*≥41*	66	13	10	15.2	0	-	0.32 ± 0.88	0.32 ± 0.88	0.5 ± 1.52	0.5 ± 1.52
			*χ*^*2*^ for trend = 4.94; p = 0.026		Fisher’s exact p = 0.001		F = 6.43; p = 0.0003	F = 4.87; p = 0.002	F = 4.35; p = 0.005	F = 4.16; p = 0.006
Child order										
*First*	123	30.9	19	15.5	4	3.3	0.46 ± 1.45	0.41 ± 1.36	0.96 ± 3.48	0.88 ± 3.44
*Second*	213	51	47	23.2	5	2.5	0.56 ± 1.4	0.54 ± 1.38	1.08 ± 3.05	0.99 ± 2.96
*Third or more*	72	18.1	12	16.7	3	4.2	0.58 ± 1.55	0.58 ± 1.55	1.07 ± 2.74	1.01 ± 2.72
			*χ*^*2*^ for trend = 0.3; p = 0.583		Fisher’s exact p = 0.683		F = 0.25; p = 0.78	F = 0.47; p = 0.627	F = 0.06; p = 0.939	F = 0.07; p = 0.935
Father’s education level										
*None/elementary school*	16	3.2	6	37.5	1	6.3	1.19 ± 2.2	1.19 ± 2.2	2.25 ± 5	2.25 ± 5
*Junior high school*	183	36.6	45	24.6	8	4.4	0.68 ± 1.6	0.66 ± 1.59	1.28 ± 3.2	1.2 ± 3.15
*High school*	246	49.2	32	13	4	1.6	0.35 ± 1.18	0.32 ± 1.09	0.74 ± 2.83	0.67 ± 2.75
*University*	55	11	13	23.6	0	-	0.42 ± 0.9	0.42 ± 0.9	0.58 ± 1.4	0.58 ± 1.4
			*χ*^*2*^ for trend = 4.52; p = 0.03		Fisher’s exact p = 0.112		F = 3.45; p = 0.016	F = 3.87; p = 0.009	F = 2.50; p = 0.059	F = 2.53; p = 0.056
Mother’s education level										
*None/elementary school*	16	3.2	5	31.3	1	6.3	0.69 ± 1.74	0.69 ± 1.74	1.19 ± 3.71	1.19 ± 3.71
*Junior high school*	141	27.8	37	26.2	8	5.7	0.83 ± 1.82	0.82 ± 1.81	1.58 ± 3.77	1.5 ± 3.72
*High school*	268	53	39	14.6	4	1.5	0.36 ± 1.14	0.33 ± 1.07	0.69 ± 2.56	0.61 ± 2.49
*University*	81	16	15	18.5	0	-	0.36 ± 0.88	0.35 ± 0.88	0.73 ± 2.07	0.72 ± 2.08
			*χ*^*2*^ for trend = 5.10; p = 0.024		Fisher’s exact p = 0.019		F = 4.11; p = 0.007	F = 4.65; p = 0.003	F = 3.08; p = 0.027	F = 3.09; p = 0.027
Maternal age at delivery, years										
*≤25*	105	20.8	33	31.4	9	8.6	1.06 ± 2.12	0.95 ± 2.03	2.05 ± 4.9	1.92 ± 4.86
*26-30*	179	35.4	31	17.3	2	1.1	0.35 ± 0.95	0.35 ± 0.95	0.75 ± 2.2	0.67 ± 2.11
*31-35*	158	31.3	23	16.5	3	1.9	0.47 ± 1.38	0.47 ± 1.38	0.85 ± 2.68	0.82 ± 2.65
*≥36*	63	12.5	8	12.7	0	-	0.24 ± 0.73	0.24 ± 0.73	0.37 ± 1.21	0.37 ± 1.21
			*χ*^*2*^ for trend = 9.36; p = 0.002		Fisher’s exact p = 0.003		F = 7.1; p < 0.001	F = 5.48; p = 0.001	F = 5.64; p = 0.001	F = 5.18; p = 0.002
***Infant feeding practices***										
History and duration of breastfeeding, months										
*No*	95	19.5	15	15.8	1	1.1	0.38 ± 1.08	0.38 ± 1.08	0.69 ± 2.1	0.69 ± 2.1
*≤ 4*	150	30.8	28	18.7	6	4	0.59 ± 1.68	0.56 ± 1.67	1.25 ± 4.05	1.19 ± 4.03
*5-10*	115	23.6	14	12.2	0	-	0.22 ± 0.62	0.22 ± 0.62	0.37 ± 1.03	0.31 ± 0.96
*11-19*	62	12.7	13	20.1	0	-	0.44 ± 1.07	0.44 ± 1.07	0.94 ± 2.47	0.76 ± 2.26
*≥ 20*	65	13.4	20	30.8	6	9.2	1.06 ± 2.08	1.03 ± 2.03	2.06 ± 4.3	2.02 ± 4.25
			*χ*^*2*^ for trend = 4.10; p = 0.043		Fisher’s exact p = 0.002		F = 4.24; p = 0.002	F = 3.95; p = 0.004	F = 3.66; p = 0.006	F = 3.75; p = 0.005
Non-nutritive sucking habits (finger or pacifier)										
*No*	473	93.8	87	18.4	14	2.9	0.51 ± 1.42	0.48 ± 1.38	1 ± 3.1	0.93 ± 3.05
*Yes*	37	7.2	9	24.3	0	-	0.49 ± 1.07	0.49 ± 1.07	0.76 ± 2.3	0.76 ± 2.3
			*χ*^*2*^ = 0.79; p = 0.374		Fisher’s exact p = 0.613		t-test = 0.09; p = 0.93	t-test = -0.02; p = 0.985	t-test = 0.47; p = 0.641	t-test = 0.28; p = 0.781
Bottle feeding										
*No*	79	15.7	19	24.1	6	7.6	0.89 ± 2.03	0.89 ± 2.03	1.84 ± 4.35	1.84 ± 4.35
*Yes*	423	84.3	77	18.2	8	1.9	0.44 ± 1.25	0.41 ± 1.2	0.84 ± 2.74	0.76 ± 2.67
			*χ*^*2*^ = 1.47; p = 0.225		*χ*^*2*^ = 7.99; p = 0.005		t-test = 2.66; p = 0.008	t-test = 2.9; p = 0.004	t-test = 2.66; p = 0.008	t-test = 2.93; p = 0.004
Sleep with sweetened bottle or pacifier										
*No*	412	80.8	71	17.2	12	2.9	0.47 ± 1.34	0.44 ± 1.3	0.9 ± 2.83	0.82 ± 2.77
*Yes*	98	19.2	25	25.5	2	2	0.67 ± 1.59	0.65 ± 1.59	1.35 ± 3.81	1.31 ± 3.8
			*χ*^*2*^ = 3.55; p = 0.06		Fisher’s exact p = 1		t-test = -1.32; p = 0.187	t-test = -1.38; p = 0.167	t-test = -1.32; p = 0.188	t-test = -1.44; p = 0.151
Start using cup, months										
*≤ 12*	284	59.9	46	16.2	7	2.5	0.42 ± 1.3	0.39 ± 1.24	0.77 ± 2.59	0.72 ± 2.56
*13-18*	58	12.2	11	19	1	1.7	0.52 ± 1.42	0.52 ± 1.42	1.34 ± 3.67	1.16 ± 3.55
*≥ 19*	132	27.9	33	25	4	3	0.64 ± 1.43	0.61 ± 1.4	1.1 ± 2.96	1.08 ± 2.92
			*χ*^*2*^ for trend = 4.44; p = 0.035		Fisher’s exact p = 0.912		F = 1.14; p = 0.321	F = 1.35; p = 0.26	F = 1.31; p = 0.27	F = 1.08; p = 0.341
Sweets consumption										
*Never*	20	4.1	1	5.0	0	-	0.05 ± 0.22	0.05 ± 0.22	0.05 ± 0.22	0.05 ± 0.22
*Less than once a day*	220	44.6	39	17.7	6	2.7	0.46 ± 1.31	0.43 ± 1.26	0.82 ± 2.56	0.77 ± 2.52
*At least once a day*	253	51.3	51	20.2	8	3.2	0.58 ± 1.52	0.56 ± 1.5	1.19 ± 3.54	1.09 ± 3.47
			Fisher’s exact p = 0.241		Fisher’s exact p = 1		F = 1.51; p = 0.222	F = 1.57; p = 0.21	F = 1.79; p = 0.168	F = 1.54; p = 0.215
***Oral hygiene habits***										
Toothbrushing habits										
*Less than once a day*	144	28.5	35	24.3	4	2.8	0.59 ± 1.49	0.57 ± 1.46	1.16 ± 3.28	1.08 ± 3.23
*Once a day*	210	41.5	32	15.2	6	2.9	0.44 ± 1.36	0.43 ± 1.34	0.85 ± 2.96	0.78 ± 2.89
*More than once a day*	152	30	31	20.4	4	2.6	0.56 ± 1.4	0.51 ± 1.33	1.07 ± 3	1.02 ± 2.98
			*χ*^*2*^ for trend = 0.67; p = 0.415		Fisher’s exact p = 1		F = 0.55; p = 0.576	F = 0.44; p = 0.647	F = 0.47; p = 0.623	F = 0.49; p = 0.612
Start toothbrushing, months										
*≤12*	76	15.8	14	18.4	3	4	0.61 ± 1.71	0.61 ± 1.71	1.28 ± 3.62	1.2 ± 3.58
*13-24*	219	45.4	33	15.1	2	0.9	0.37 ± 1.09	0.34 ± 1.02	0.73 ± 2.32	0.64 ± 2.22
*≥25*	187	38.8	42	22.5	7	3.7	0.61 ± 1.48	0.58 ± 1.44	1.1 ± 3.26	1.05 ± 3.23
			*χ*^*2*^ for trend = 1.57; p = 0.21		Fisher’s exact p = 0.087		F = 1.91; p = 0.15	F = 2.11; p = 0.122	F = 1.31 p = 0.271	F = 1.52; p = 0.221
Toothpaste use										
*No*	32	6.4	3	9.4	2	6.3	0.47 ± 1.74	0.47 ± 1.74	0.93 ± 3.83	0.93 ± 3.83
*Yes*	467	93.6	91	19.5	12	2.6	0.52 ± 1.39	0.49 ± 1.35	1 ± 3.02	0.93 ± 2.97
			Fisher’s exact p = 0.24		Fisher’s exact p = 0.224		t-test = -0.19; p = 0.848	t-test = -0.09; p = 0.925	t-test = -0.12; p = 0.906	t-test = 0.01; p = 0.991
Adult supervision of toothbrushing										
*No*	26	5.3	5	19.2	2	7.7	0.81 ± 2	0.81 ± 2	1.62 ± 4.61	1.62 ± 4.61
*Yes*	466	94.7	85	18.2	10	2.2	0.47 ± 1.3	0.44 ± 1.26	0.9 ± 2.79	0.83 ± 2.72
			*χ*^*2*^ = 0.02; p = 0.899		Fisher’s exact p = 0.128		t-test = 1.24; p = 0.214	t-test = 1.38; p = 0.169	t-test = 1.22; p = 0.224	t-test = 1.37; p = 0.171
Use of fluoride supplements										
*No*	314	64.3	58	18.5	7	2.2	0.48 ± 1.34	0.45 ± 1.29	0.92 ± 2.83	0.84 + 2.76
*Yes*	174	35.7	34	19.5	7	4	0.6 ± 1.57	0.59 ± 1.54	1.2 ± 3.56	1.15 ± 3.53
			*χ*^*2*^ = 0.08; p = 0.772		*χ*^*2*^ = 1.29; p = 0.256		t-test = -0.93; p = 0.351	t-test = -1.04; p = 0.297	t-test = -0.94; p = 0.35	t-test = -1.08; p = 0.281
***Access to dental services***										
Dental visit in the previous year										
*No*	413	83.1	62	15	6	1.5	0.35 ± 1.1	0.34 ± 1.1	0.64 ± 2.28	0.59 ± 2.23
*Yes*	84	16.9	33	39.3	8	9.5	1.37 ± 2.24	1.25 ± 2.17	2.82 ± 5.2	2.63 ± 5.17
			*χ*^*2*^ = 26.6; p < 0.001		*χ*^*2*^ = 16.61; p < 0.001		t-test = -6.27; p < 0.001	t-test = -5.68; p < 0.001	t-test = -6.13; p < 0.001	t-test = -5.8; p < 0.001

ECC = early childhood caries, S-ECC = severe early childhood caries.

As regards to social demographics, age of child, age of the mother at the time of the survey and at time of delivery, and education level of both parents were all significantly associated to ECC pattern. Indeed, at univariate analysis, ECC prevalence significantly increased with age of the child, ranging from 8.7% in 3 years old children to 29.8% in 5 years old children (*χ*^*2*^ for trend = 22.89; p < 0.001), whereas it significantly decreased with increasing age of the mother at the time of the survey, ranging from 30.5% in mothers ≤ 30 years to 15.2% in ≥ 41 years of age (*χ*^*2*^ for trend = 4.94; p = 0.026) and at the time of delivery, ranging from 31.4% in mothers delivering at 25 or younger to 12.7% in those older than 35 at delivery (*χ*^*2*^ for trend = 9.36; p = 0.002); the same significant decreasing trend of ECC prevalence was found for education level of mothers, ranging from 31.3% in those with no more than elementary school degree to 18.5% in those with a university degree (*χ*^*2*^ for trend = 5.1; p = 0.024), and for education level of fathers (*χ*^*2*^ for trend = 4.52; p = 0.03). No significant differences were found according to gender (*χ*^*2*^ = 0.87; p = 0.352), father’s age (*χ*^*2*^ for trend = 0.18; p = 0.676), and child order (*χ*^*2*^ for trend = 0.3; p = 0.583). Some of the infant feeding practices, such as history and duration of breastfeeding, having experienced sleep with sweetened bottle or pacifier, and time of onset of cup use appear to be related to ECC experience, since ECC prevalence significantly increased from 15.8% in non breastfed subjects to 30.8% in children who were breastfed for 20 months or longer (*χ*^*2*^ for trend = 4.1; p = 0.043), from 17.2% in those who did not have the habit to go to sleep with sweetened bottle or pacifier to 25.5% in those who did (*χ*^*2*^ = 3.55; p = 0.06), and from 16.2% to 25% with increasing age when children started using cups (*χ*^*2*^ for trend = 4.44; p = 0.035). The other feeding/eating practices investigated, that is non-nutritive sucking habits with finger and/or pacifier (*χ*^*2*^ = 0.79; p = 0.374), bottle feeding (*χ*^*2*^ = 1.47; p = 0.225) and frequency of sweets consumption (Fisher’s exact p = 0.241) did not show any significant association with ECC. Analogously, none of the oral hygiene habits taken into account, such as frequency (*χ*^*2*^ for trend = 0.67; p = 0.415), time of onset (*χ*^*2*^ for trend = 1.57; p = 0.21), and adult supervision of toothbrushing (*χ*^*2*^ = 0.02; p = 0.899), as well as toothpaste (Fisher’s exact p = 0.24) and fluoride supplements (*χ*^*2*^ = 0.08; p = 0.772) use, seems to influence significantly the risk of ECC in our sample. By contrast, having been visited by a dentist in the previous year is associated with a more than doubled prevalence of ECC, compared to those who did not have access (*χ*^*2*^ = 26.6; p < 0.001).

The associated factors for mean dmft almost resembled those observed for ECC; however, in some instances, although the trend was similar, statistical significance was different. This was the case for bottle feeding, sleep with a sweetened bottle and/or pacifier, and age at the start of cup use. In particular, mean dmft was significantly lower in children who had experienced bottle feeding (t-test = 2.66; p = 0.008), whereas it was not significantly higher in children who used to sleep with sweetened bottle or pacifier (t-test = -1.32; p = 0.187) and in those who started to use cups later (F = 1.14; p = 0.321).

Results on prevalence of S-ECC stratified by several characteristics appear less stable compared to ECC, due to the exiguous number of events. However, a pattern similar to that of ECC was found for most characteristics, although, for the low number of events, statistical significance was not always found. S-ECC increased with increasing age of child, and decreased with increasing age of the mother at the time of the survey and at time of delivery, and decreased with increasing education level of both parents; however, only for mother’s age at the time of the survey (Fisher’s exact p = 0.001) and at time of delivery (Fisher’s exact p = 0.003), and for mother’s education level (Fisher’s exact p = 0.019), the differences were statistically significant at univariate analysis. Among the infant feeding practices, severity of ECC was significantly related to longer duration of breastfeeding (Fisher’s exact p = 0.002), whereas, unexpectedly, S-ECC was significantly lower (1.9%) in bottle fed children, compared to those who were not (7.6%) (*χ*^*2*^ = 7.99; p = 0.005). Among the explored oral hygiene habits, no substantial differences were found according to severity of ECC, except for toothbrushing adult supervision, since S-ECC ranged from 7.7% in no supervised children to 2.2% in the supervised ones, although this difference was not statistically significant at the univariate analysis (Fisher’s exact p = 0.128). Finally, S-ECC was unevenly distributed according to visits by a dentist in the previous year, ranging from 1.5% in those who were not visited to 9.5% in those who did (*χ*^*2*^ = 16.61; p < 0.001).

Results from the logistic regression models showed that prevalence of ECC significantly increased with age of the child (OR = 1.95; 95% CI = 1.3-2.91), and duration of breastfeeding (OR = 1.26; 95% CI = 1.01-1.57), whereas it was significantly lower in children of more educated mothers (OR = 0.64; 95% CI = 0.42-0.96), and significantly higher in those who had been visited by a dentist in the previous year (OR = 3.29; 95% CI = 1.72-6.33) (Model 1 in Table 2). A similar pattern was found for S-ECC, that significantly increased with breastfeeding (OR = 2.06; 95% CI = 1.13-3.76), was significantly lower with increasing mothers’ education (OR = 0.16; 95% CI = 0.05-0.54), and was higher in children who had visited a dentist (OR = 5.54; 95% CI = 1.15-26.66), whereas no significant association was found with children’s age (Model 2 in Table 2).

**Table 2:** Results of the logistic regression models

	**OR**	**SE**	**95% CI**	**p**
**Model 1. Outcome: ECC**				
Log-likelihood = -166.48, χ^2^ = 47.68, p < 0.001				
Dental visit in the previous year	3.29	1.1	1.72-6.33	<0.001
Age	1.95	0.4	1.3-2.91	0.001
Mother’s education level	0.64	0.13	0.42-0.96	0.032
History and duration of breastfeeding	1.26	0.14	1.01-1.57	0.039
Start using cup	1.29	0.21	0.95-1.77	0.103
Sleep with sweetened bottle or pacifier	1.63	0.55	0.84-3.16	0.152
Start toothbrushing	1.16	0.25	0.76-1.77	0.497
Maternal age at delivery	0.84	0.3	0.42-1.71	0.637
Mother’s age	0.99	0.36	0.49-2.03	0.998
**Model 2. Outcome: S-ECC**				
Log-likelihood = -31.77, χ^2^ = 22.9, p = 0.0063				
Mother’s education level	0.16	0.1	0.05-0.54	0.003
History and duration of breastfeeding	2.06	0.63	1.13-3.76	0.019
Dental visit in the previous year	5.54	4.44	1.15-26.66	0.033
Sleep with sweetened bottle or pacifier	0.61	0.7	0.06-5.93	0.667
Mother’s age	1.34	1.22	0.23-7.97	0.748
Start using cup	1.09	0.46	0.47-2.51	0.839
Age	0.91	0.46	0.34-2.43	0.851
Start toothbrushing	1.04	0.56	0.36-2.98	0.938
Maternal age at delivery	0.3	0.27	0.05-1.81	0.188

ECC = early childhood caries, S-ECC = severe early childhood caries.

## Discussion

This survey was intended to investigate ECC pattern and severity in a large sample of children in an area in Southern Italy, and to identify factors that may be related to this condition, and that may become the focus for interventions aimed at the prevention of ECC.

Our findings revealed that ECC still represents a consistent burden in our population, interesting almost 20% of 3–5 years old children, whereas almost 3% are affected by the more severe disease. In the vast literature aimed at the exploration of ECC prevalence, studies conducted in Europe [40-45] have reported prevalence of ECC ranging from 11.4% in 3–6 years old Swedish children [46] to 55.1% in 5 years old children in the Czech Republic [47]. In Italy, reported prevalence of ECC ranged from 8% [48] to 31.6% [49], and our figure are similar to many other European and Italian studies [43,48-54]. Only few studies have analysed S-ECC, and a prevalence of 6.5% has been reported in 3 years old children in Lithuania [55], and of 12.2% in 2–3 years old children in Sweden [41]. It should be pointed out, however, that these studies have been conducted with differing age ranges, methods, definitions of ECC, and time frame, and therefore, comparisons must be interpreted cautiously.

As in most studies aimed at the investigation of the oral health of pre-school children, we found a high proportion of caries-free subjects and a mean dmft of 0.5, whereas the pattern and severity of caries are polarized to certain subgroups of the population resembling their socioeconomic status [7,40,52]. Moreover, in children with caries the dmft tends to be high, reaching a mean of 6.86 in those with S-ECC. Indeed, when we analyzed the role of several conditions that are known or controversial factors related to ECC pattern and severity, we found that ECC was not evenly distributed in the population, but it is substantially influenced by social demographics and infant feeding practices: although the overall picture on prevalence of ECC and S-ECC does not appear particularly concerning, a more detailed description of this phenomenon through stratification according to several characteristics, shows significant differences across subgroups of the population. This circumstance is alarming, considering that most differences, as reported in previous studies, are related to social demographics [8,12,15,20] and infant feeding practices [18,19], and therefore, place for improvement is substantial. The finding that the overall ECC prevalence of about 20%, in line with with the 80% caries free at age 6 target set by WHO within the Health21 policy framework [56], becomes about 30% in older (5 years) children, in children born from younger mothers at the time of survey and of delivery, and who had a lower education level poses attention on the role that social and cultural factors may have on the occurrence of ECC, delineating dramatic differences across social groups. Analogously, the difference of about 6–10 points compared to the overall ECC prevalence according to some feeding practices, underscores the need for more effective interventions to promote prevention in the field of oral health in infancy. In particular, since the significantly higher prevalence was found in children who were longer breastfed, who used to go to sleep with sweetened bottle or pacifier and later began to drink from cups, it appears that a strategic role in the prevention of ECC may be played by pediatricians, since they are the first health care professionals who are contacted by mothers, and represent the reference figure for counseling on feeding practices. This acquires even more substance in the Italian context, since every newborn is assigned a pediatrician free of charge within the NHS, that represents the “gate keeper” for all other accesses to health care within the NHS. However, a recent study conducted by one of us on knowledge, attitudes, and practices of pediatricians regarding prevention of oral diseases in Italy raised the issue of lack of knowledge of the main associated factors for oral diseases, although almost all pediatricians believed that they had an important responsibility in preventing oral diseases in children and provided an oral examination on their patients [57]. Moreover, the statistical association between breastfeeding duration and ECC must be interpreted cautiously since it is important to consider that the present study included children who were no longer breastfed at the time of examination. This may have biased results as caries presence 3–4 years after breastfeeding has ceased might not be attributed to breastfeeding. Moreover, it should be considered that exclusive breastfeeding is only performed during the first 6 months of life, and subsequent introduction of new foods, especially those rich in sucrose, is a confounding factor when analyzing the association between breastfeeding and caries [31].

Moreover, the finding that children with ECC and S-ECC are significantly more likely to have visited a dentist in the previous year suggests that dentists are contacted only after ECC has occurred; therefore, it seems that dentists do not play an effective role in the prevention practices.

Unexpectedly, we did not find an association between ECC or S-ECC and bottle feeding, whereas it is well-known that particularly severe disease has long been called “nursing caries” or “bottle caries”. However, we may hypothesize that it is not bottle feeding per se that represents an associated factor for ECC and S-ECC, but, more consistently, the consumption, through nursing bottles, of sweetened liquids, and indeed, our study clearly showed the importance of sweetened bottle or pacifier, particularly at night, as possible associated factors for ECC and S-ECC.

It is well-known that oral hygiene practices are among the most effective measures in the prevention of dental caries, and we found that only toothbrushing adult supervision was associated with a significantly lower risk of S-ECC. This is not surprising, since in young children oral hygiene is almost completely a parents’ responsibility, whereas simple assessment of frequency of tooth brushing may not be a valid indicator of oral hygiene.

### Limitations

We used a self-administered questionnaire to register all potential associated factors. This could have determined a shift to more socially desirable answers, even though this does not appear to be the case in our study, since even self-reporting demonstrated a wide-spread diffusion of associated factors in our population. Moreover, the cross-sectional nature of the study does not allow any cause-effect relationship, since data on “associated factors” and “outcomes” are assessed at the same time. Nonetheless, this study represents a useful way to determine the prevalence of ECC and S-ECC and, eventually, to identify differences among subgroups disaggregated by social demographics, infant feeding practices, oral hygiene habits, and access to dental services, in order to target preventive interventions on those subjects that manifest poorer oral health. As with any survey based on a self-administered questionnaire, information resulting from the memory of parents may not be entirely accurate, primarily because of the long time frame used in the study that may have introduced recall bias. On the other hand, longer time frames are useful for formulating broad prevalence estimates in a context in which no data are available. Finally, problems regarding representativeness and generalizability should be taken into account, and we believe that the sampling methods and the high response rate allows us to be confident on the representativeness of the chosen sample and that our results may be generalized to children living in the Southern part of Italy.

## Conclusions

In conclusion, results of our study demonstrate that even in western countries ECC and S-ECC still represent a significant burden in preschool children, particularly in those disadvantaged, and that most of the known modifiable associated factors regarding feeding practices and oral hygiene are still very spread in the population. Paediatricians and dentists should play a more effective role in the prevention of ECC.

## Competing interests

The authors declare that they have no competing interests.

## Authors’ contributions

CGAN and MP designed and coordinated the study. LF performed dental examinations. AB, LF and CP contributed to the data analysis and results interpretation. MP wrote the manuscript. All authors read and approved the final manuscript.

## Pre-publication history

The pre-publication history for this paper can be accessed here:

http://www.biomedcentral.com/1471-2458/14/206/prepub
